# Chronic Ingestion of High Dosed Phikud Navakot Extraction Induces Mesangiolysis in Rats with Alteration of AQP1 and Hsp60 Expressions

**DOI:** 10.1155/2015/462387

**Published:** 2015-03-01

**Authors:** Kanchana Kengkoom, Sumate Ampawong

**Affiliations:** ^1^Academic Services Office, National Laboratory Animal Center, Mahidol University, 999 Salaya, Puttamonthon, Nakorn Pathom, Bangkok 73170, Thailand; ^2^Department of Tropical Pathology, Faculty of Tropical Medicine, Mahidol University, 420/6 Ratchawithi Road, Ratchathewi, Bangkok 10400, Thailand

## Abstract

Phikud Navakot (PN) is commonly used in Thai traditional medicine for alleviation of cardiovascular and cerebrovascular symptoms; however little is known about the chronic toxicity effects of the extracts from the herbs in PN. Repeated extraction doses of 10, 100, and 1,000 mg/kg/day were randomly administered to both male and female Sprague Dawley rats for 12 months. Histopathological study revealed that mesangiolysis was predominately found at the highest dose. Aquaporin 1 (AQP1) expression in the mesangiolytic glomeruli was significantly lower than in the intact glomeruli. This may be relevant to an imbalance of vascular function manifested by AQP1 alteration. In the mesangiolytic glomeruli, 60 kDa heat shock protein (Hsp60) was significantly upregulated on the endothelial lining cells of aneurysm and vascular cyst. Hsp60 increase may be related to endothelial cell damage due to its intracellular protective role. Blood urea nitrogen and creatinine levels remained within their normal range indicating well-functioning renal reserve function. In conclusion, high dosed PN may affect the endothelium leading to inability of vascular permeability and consequence to mesangiolysis. Our results suggest that only a high dose of chronic oral administration of PN is relatively toxic in association with mesangiolysis. The NOAEL was determined to be 100 mg/kg/day.

## 1. Introduction

Mesangiolysis is a harmful glomerular process leading to necrosis of mesangial cells and/or endothelial cells within the glomeruli. Several conditions are known causes of mesangiolysis, such as toxic glomerulopathy from Habu snake venom [1], circulatory disturbance from congenital heart disease [2], microangiopathy from hemolytic uremic syndrome [3], glomerulonephritis from anti-thymocyte-1 antibody [4], focal glomerulosclerosis [5], transplantation [6], radiation [7], diabetes mellitus [8, 9], amyloidosis [10], and monoclonal Ig deposition disease [11]. However, mesangiolysis is rarely found in preclinical toxicity studies [12], and herb extraction induced mesangiolysis has not yet been reported.

Herbal and natural products are commonly prescribed as alternative medicine in developing countries especially Thailand. Like Phikud Navakot (PN), major ingredients of Yahom Navakot are composed of nine Thai herbal plant species,* Anacyclus pyrethrum*,* Angelica dahurica*,* Angelica sinensis*,* Atractylodes lancea*,* Artemisia annua*,* Ligusticum sinense*,* Picrorhiza kurroa*,* Saussurea lappa*, and* Terminalia chebula*. PN has a long tradition of use in Thailand to alleviate hyperlipidemia, cardiovascular diseases, cerebrovascular diseases, asthma, diabetes mellitus, and some kinds of cancer [13–20]. However the purified extraction of this herb has not been investigated for chronic oral toxicity. Therefore, repeated doses of its aqueous extracts were evaluated in Sprague Dawley rats. At the end of the study, hematology, blood clinical chemistry, and histopathology studies were examined.

In addition, to identify the pathogenesis of considered histopathological changes especially mesangiolysis, two proteins thought to be involved in this mechanism were selected for immunohistochemical study. Aquaporins (AQPs), membrane-inserted water channel proteins, play a highly important role in the reabsorption of water and homeostasis of the vascular system [21]. 60 kDa heat shock protein (Hsp60), mitochondrial chaperone, is involved in stress response and apoptosis inhibition [22, 23]. The results of this study provide new insights into the effects and the precautions required to appropriately consume PN.

## 2. Materials and Methods

### 2.1. Animal Husbandry

Animal studies were performed in accordance with the Mahidol University policy for the care and use of animals for scientific purposes and approved by the institutional animal ethics committee (Animal Welfare assurance number: RA2011.03). Healthy eight-week-old Sprague Dawley rats from the National Laboratory Animal Center, Mahidol University, Thailand, were used. All rats were housed in an environment of 23 ± 2°C, 55 ± 15% relative humidity, 10–15 air change per hour ventilation, and 12:12 hours of dark and light cycle and provided with pasteurized standard diet and 7–10 ppm chlorinated water* ad libitum*.

### 2.2. Test Article

The extracts of PN were kindly prepared by Associated Professor Dr. Uthai Sotanaphun, Department of Pharmacognosy, Faculty of Pharmacy, Silpakorn University, Nakorn Pathom, Thailand. Raw materials of the nine herbs were mixed and ground into a powder. The powder was immersed in 80% ethanol overnight, then boiled for 3 hours, and filtered to remove the residue. Next, the aqueous extracts were repeatedly boiled for 3 hours and filtrated. The aqueous extracts were spray-dried to remove trace solvent.

### 2.3. Chronic Toxicity Test

Male and female rats were administered daily oral doses of 10, 100, and 1,000 mg/kg of the extract for 12 months (*n* = 10 rats/group) while the control group was given water at the same volume as the tested extracts. During the period of the study, all rats were daily observed for mortality and clinical signs of toxicity.

### 2.4. Hematology and Blood Clinical Chemistry

At the end of the study, the rats were euthanized by overdose inhalation of carbon dioxide. Blood samples were collected by cardiac puncture and rats were humanly killed by exsanguinations. Hematological and blood clinical chemistry tests were conducted by an ABBOTT CELL-DYN 3500 system (ABBOTT Laboratories, IL, USA) and a Hitachi 902 automated blood analyzer (Hitachi Science Systems Ltd., Ibaraki, Japan).

### 2.5. Histopathology

All tested rats were subjected to gross necropsy. All gross pathological changes were microscopically examined. The heart, liver, lung, kidney, and spleen were removed and then were fixed in 10% neutral buffer formalin. Fixed specimens underwent standard processing and were embedded in paraffin wax. Sections (5 *µ*m) were mounted on glass slides for staining by hematoxylin and eosin (H&E). All instances of histopathological change were examined. Overall histopathological appearances were scored as 0 = absent, +1 = mild (<25%), +2 = moderate (25–50%), and +3 = severe (>50%).

**Figure 1:**
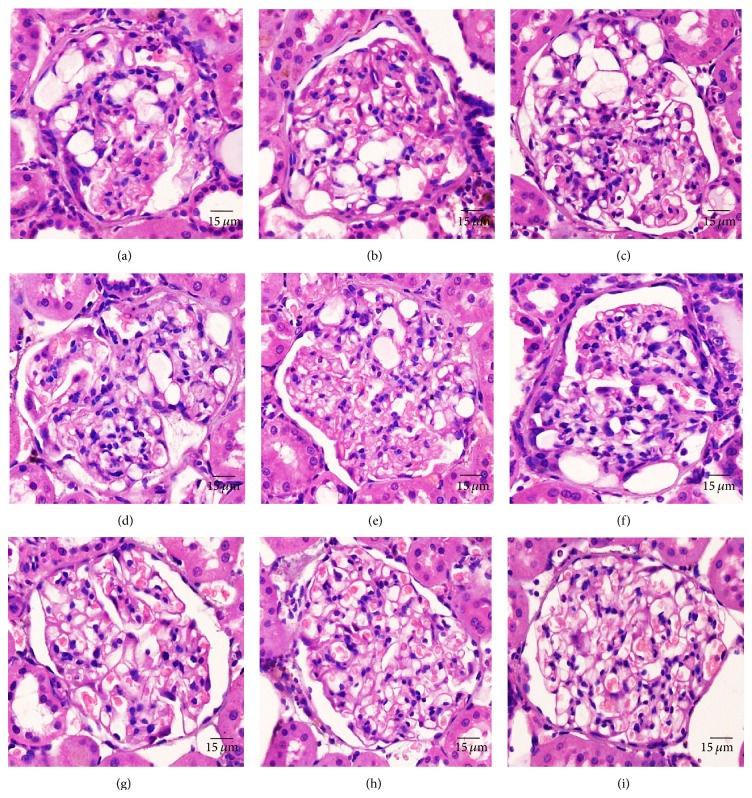
The severity score of mesangiolysis; H&E staining of mesangiolytic glomeruli indicated the severity as mild (+1) (g)–(i), moderate (+2) (d)–(f), and severe (+3) (a)–(c) depending on the degree of vascular and endothelial cell damage.

For mesangiolysis, the glomerulus was examined at least 50 glomeruli/rat then the prevalence of mesangiolysis was calculated. The severity was simultaneously scored as follows: 0 = absent, +1 = mild glomerular endothelial swelling to generalized capillary aneurysm (Figures 1(g)–1(i)), +2 = developed glomerular cyst which is <50% of glomerular area (Figures 1(d)–1(f)), and +3 = developed glomerular cyst which is >50% of glomerular area (Figures 1(a)–1(c)).

### 2.6. Immunohistochemistry

Polyclonal rabbit antiaquaporin 1 (AQP1) (Millipore, USA, AB3272-200UL) and polyclonal rabbit anti-60 kDa heat shock protein (Hsp60) (Bioss, USA, 900291W) were used as indicators of the vascular system and endothelial cell anomaly. Five micron thick sections from the paraffin blocks were cut and placed on precoated immunohistochemistry slides, then dried overnight at 56°C, and then allowed to cool. The sections were deparaffinized in xylene and rehydrated prior to immunostaining. Heat-induced antigen retrieval with citrate buffer (pH 6) was used to unmask the antigen from all antibodies. Endogenous peroxidase was quenched with 3% v/v hydrogen peroxide in methanol after sections were cooled. The sections were washed with 0.2% v/v Tween in phosphate buffered saline (PBS) and blocked with protein block serum-free (Dako, Denmark, X0909) for 10 min. Sections were incubated in primary antibody diluted in PBS with 1% v/v normal goat serum (NGS, Vector, USA, S1000). The sections were washed and incubated at room temperature for 30 min with labeled polymer HRP anti-mouse/rabbit EnVision kit (Dako, Denmark, K5007) and visualized with diaminobenzidine (DAB, Dako, Denmark, K3468). The slides were counterstained with hematoxylin before permanent mounting with Permount.

Fifty intact and mesangiolytic glomeruli were randomly examined and images were captured at 400x magnification. Immunohistochemical expression of AQP1 and Hsp60 was then analyzed using ImageJ, NIH [24]. Color images were first converted to 8 bits in gray scale. Adjusted images were transformed by threshold mode to locate the area of interest. The polygon selection mode was used to draw a line over the area of glomerulus. The area of positive reaction was estimated by the number of black pixels with measure mode as percentage area of expression/glomerular area.

### 2.7. Statistical Analysis

Quantitative results were expressed as mean ± standard deviation. Qualitative results were expressed as prevalence. Data were statistically analyzed with IBM SPSS statistical software version 20. One way analysis of variance (ANOVA) and Levene's test were used to differentiate the difference of mean and variance among groups. Multiple comparison, Bonferroni test, or Dunnett test was performed for equal and nonequal variance assumption. Pearson Chi-square test was used to differentiate the proportion among groups.

## 3. Results

### 3.1. Survival and Clinical Signs

76 rats (total *n* = 80) survived to the end of the study. The cause of death was considered to be related to gavage technical error which was histopathologically presented by aspirated pneumonia. There was no toxicity-related mortality. Treatment and concurrent control groups were similar in clinical manifestations.

### 3.2. Hematology and Blood Clinical Chemistry

Mean hematological and blood clinical chemistry parameters are shown in Tables 1 and 2. All of these parameters in both treatment and concurrent control groups were similar. No remarkable changes were observed in hematologic and clinical chemistry parameters.

### 3.3. Histopathology

**Figure 2:**
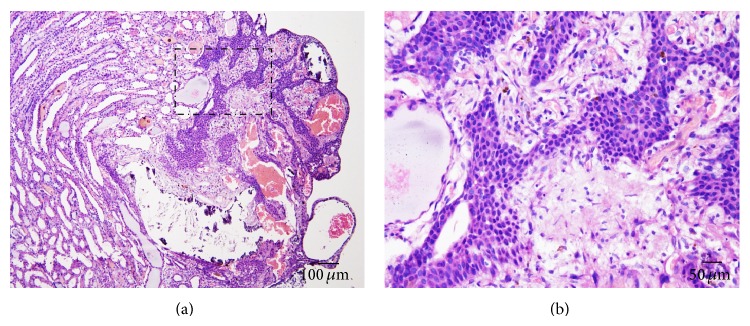
Transitional cell hyperplasia; H&E staining of renal pelvis urothelium which showed an increased number of urothelial cells without cellular atypia (uniform cell arrangement), characterized by nodular growing pattern as exophytic (protruding outward to lumen), and presented with moderate to severe calcification; (b) is higher magnification of (a)'s inset.

There was no toxicity-related gross in the hearts, livers, lungs, and spleens at any tested dosages. Histopathological findings are shown in Table 3. Most were similar prevalence when comparing the treatment and concurrent control groups. In the kidney, we found toxicity-related lesion at the highest dose of PN. Both sides of the kidney exhibited the prevalence of mesangiolysis which significantly differed from those of 10, 100 mg/kg/day dosages and the control group. However its prevalence did not vary according to side or sex of any group. The mean severity score in all groups was also similar. The lesions were ranked from dissolution of the mesangial cells, glomerular endothelial swelling, fully developed capillary aneurysm (Figures 1(g)–1(i)), and glomerular cyst (Figures 1(a)–1(f)). A glomerular cyst is a large vascular space arising by the merger of the dilated mesangium and adjoined capillary. Microangiopathy, glomerulosclerosis, glomerulonephritis, amyloidosis, lamellate nodule, fibrin deposit, and leukocyte accumulation were not seen in these mesangiolytic glomeruli. Additional notable lesions related to nontreatment-related response were pelvis calcification and transitional cell hyperplasia (Figure 2) which were significantly higher in the control group than the others (Table 3).

### 3.4. Immunohistochemistry

**Figure 3:**
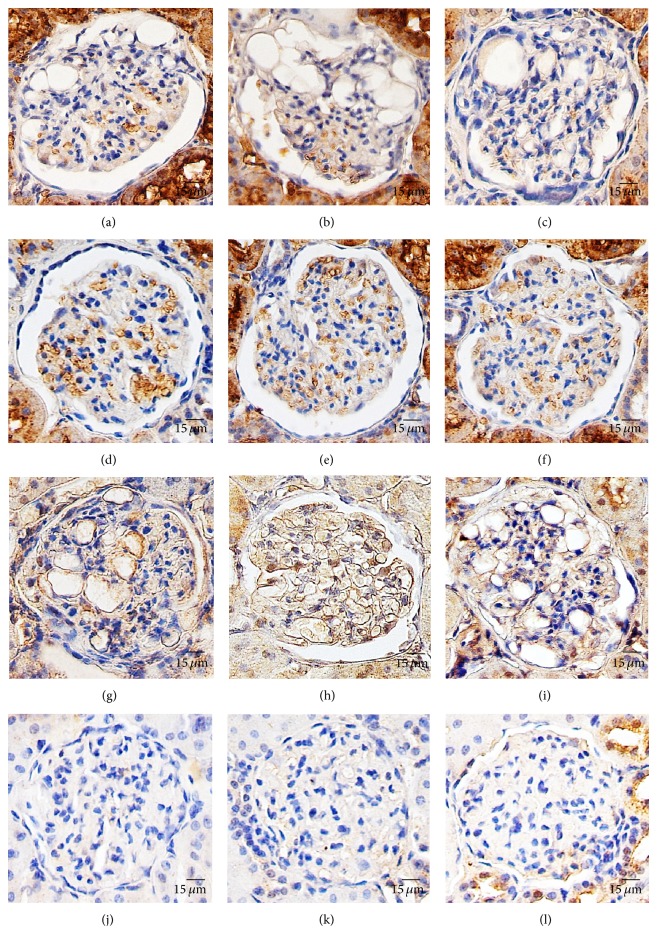
AQP1 and Hsp60 expression in the glomeruli; immunohistochemical staining exhibited AQP1 and Hsp60 expression in the mesangiolytic glomeruli ((a)–(c) and (g)–(i), resp.) and the intact glomeruli ((d)–(f) and (j)–(l), resp.). AQP1 was expressed on red blood cells in the glomeruli, while Hsp60 was expressed on the endothelial lining cell.

In general, AQP1 is expressed on endothelial cells [25–27], red blood cells, and proximal tubules [28]. Hsp60 is expressed on both the renal cortex and medulla [29], particularly in the cytoplasm and mitochondria of any cell [30]. In this study, we compared their expression in the glomeruli and our results indicate that AQP1 was expressed on red blood cells (Figures 3(a)–3(f)). In the mesangiolytic glomeruli, Hsp60 was highly expressed on endothelial lining cells of the capillary aneurysm (Figure 3(h)), large aneurysm, and glomerular cyst (Figures 3(g) and 3(i)) while the expression on intact glomeruli was faint (Figures 3(j)–3(l)).

The image analysis demonstrated that AQP1 expression in the mesangiolytic glomeruli was significantly lower when compared to the intact glomeruli (1.45 ± 0.01% and 8.93 ± 0.09%, *P* value = 0.004). In contrast to Hsp60, the expression in the mesangiolytic glomeruli was significantly higher than in intact glomeruli (13.76 ± 0.02% and 0.65 ± 0.01%, *P* value = 0.000).

## 4. Discussion

PN is listed in the National Public Health Ministry of Thailand's list of herbal medical products. Our previous studies have revealed the efficacy and safety of PN. An experimental study demonstrated that PN reduces dizziness and fainting due to its vasorelaxation property [31], increases mean arterial and diastolic blood pressure, and increases tail blood flow [32].* In vitro* studies also demonstrated that PN ameliorates endothelial stress due to its synergistic antioxidant property [33], attenuates oxidative stress-induced apoptotic cell death, inhibits platelet aggregations, and relates to glucose catabolism [32]. Acute and subchronic toxicity studies revealed that single and repeated oral administration of PN is relatively nontoxic with high dosage contraindication for serum uric acid elevation [34]. The present study demonstrated chronic oral toxicity effects of the extracts from herbs in PN. In each group, hematology and blood clinical chemistry values remained within the normal range. A histopathological study revealed that almost all observed lesions in liver, heart, lung, spleen, and kidney were not related to toxicological effect from PN. Rather, they were associated with physiological changes, aging lesions, and oral gavage induced lesions [24]. The only histopathological change related to toxicological effect from chronic ingestion of high dosed PN is mesangiolysis.

Based on mode of origin and morphological features, there are three types of recognizable mesangiolysis [35]. The first is the severe form with glomerular cyst formation indicating primary mesangial injury [36]. The second type is characterized by extensive widening of subendothelial space as a consequence to endothelial injury. The third type is associated with lamellate nodules caused by persistent endothelial and mesangial cell damage. In this study, the mesangiolysis caused by chronic oral administration of high-dosed PN is likely type 1 and 2 depending on morphology. The histopathological study revealed that half of the counted glomeruli showed two patterns of mesangiolysis, which is characterized by small to large aneurysm and cystic mesangiolysis. The glomerular architecture did not show evidence of thrombotic microangiopathy, glomerulonephritis, or glomerulosclerosis.

The mechanism of mesangiolysis has been discussed in many studies relevant to marked inability of vascular permeability, local intravascular coagulation, or direct effect on the mesangial cells [37]. Examples of mesangiolysis due to primary mesangial injury are Habu venom nephropathy [1], anti-thymocyte-1 antibody [4], and mitomycin C [37]. Other conditions related to endothelial cell injury are secondary mesangiolysis [2, 3, 5, 7–11]. One study postulated the mechanisms of mesangiolysis are both direct and indirect. Glycol ethers could be a direct toxicity on mesangial cells or indirect toxicity leading to hemolysis and possibly microangiopathy [38].

AQP1 in red blood cells is downregulated in some renal diseases, which is relevant to the imbalance of the vascular system and endothelial cells such as uremic syndrome [39]. Unfortunately, there have been no reports about AQP1 alteration related to glomerular diseases in rats. However, our study showed a reduction of AQP1 immunolabelling in the mesangiolytic glomeruli. This decline is characterized by decreasing numbers of glomerular red blood cells with normal expression on their surface. This reduction may indicate an inability of vascular permeability and glomerular circulation due to vascular defects which correlate to aneurysm and cystic formation in the mesangiolytic glomeruli.

There are many reports describing the relationship between the role of Hsp60 and kidney diseases. Generally they conclude that overexpression of Hsp60 enhances cytoprotective function and is induced by cellular stress processes such as chronic kidney disease [40], diabetic nephropathy (hyperglycemia and glomerular hypertension) [41], and renal failure [42]. These studies concluded that Hsp60 expression usually increases in mesangial cells and podocytes, probably due to their susceptibility to injury.

AQP1 alteration is the most important evidence from our study to support that PN induced mesangiolysis may be associated with vascular permeability imbalance or endothelial cell alteration. This is in agreement with upregulation of Hsp60 on endothelial lining cells of aneurysm and vascular cyst which indicate an injury or stress condition. Like those mentioned studies, high dosed PN may affect the endothelium and its closet neighbor cells, leading to mesangiolysis.

In addition, toxicological studies of herbs that affect glomeruli have been limited. Few reports investigate the adverse effects of herbs on renal tissues, for instance, interstitial edema and tubular necrosis in* Phyllanthus amarus* toxicity [43], high dosed* Labisia pumila* induces multiple foci of necrosis and focal pyelitis [44], and Chinese-herb containing aristolochic acid enhances urothelial atypia and transitional cell carcinoma [45]. Therefore, this study is the first report highlighting that extracts from herbs in PN affect glomeruli and can induce mesangiolysis.

The association between renal phenotypes of blood chemistry and mesangiolysis is limited. Most of the study reported that blood urea nitrogen (BUN) and creatinine levels were markedly raised in mesangiolysis with other pathological lesions, particularly advanced or end-stage renal diseases, endarteritis like lesions, cellular proliferation, glomerular enlargement, and endothelial or mesangial cell swelling [46–48]. In our study, both hematological and clinical chemistry values stayed within the normal range even with 1,000 mg/kg/day dosages including BUN and creatinine. These indicate that renal function is still preserved. It showed that mesangiolysis alone, without any pathological change, cannot alter renal function parameters.

One more interesting pathological finding in this study is transitional cell hyperplasia which was not considered to be toxic-related response. It is noted that urothelial or transitional cell hyperplasia is an important pathologic appearance of urothelial change in renal papillae which occurs as a response to bacterial infection, urinary tract toxins, carcinogens, and calculi and in association with renal papillary necrosis [12]. In the present study, transitional cell hyperplasia in the control rats was correlated to high prevalence of renal pelvis calcification (Table 3) which may relate to their physiological changes.

## 5. Conclusions

In conclusion, this work evaluates chronic toxicity of extracts from nine herbs in PN. The investigation demonstrated that they are relatively safe, as they did not cause any lethality nor produced any remarkable physiological, behavioral, hematological, blood clinical chemistry, and anatomical adverse effects of chronic toxicity in rats. The NOAEL was determined to be 100 mg/kg/day. However the contraindication of the prolonged usage for high dose oral administration should be considered remarkable mesangiolysis.

## Figures and Tables

**Table 1:** Sprague Dawley mean hematological value in chronic oral toxicity test.

Group	WBC (10^3^ *µ*L)	RBC (10^6^ *µ*L)	HCT (g/dL)	MCV (%)	MCH (fl)	MCHC (pg)	PLT (g/dL)	RDW (10^5^ *µ*L)	PDW (%)	MPV (fl)	PCT (fl)	Differential count (%)				
												(%)	Nuet	Lymph	Eosi	Mono
Male																
Control																
Mean	4.32	8.01	15.29	43.46	54.32	19.12	35.19	910.93	18.01	17.71	7.65	0.70	25.14	65.95	1.55	0.00
SD	2.12	0.36	0.61	1.38	1.10	0.72	1.27	91.15	0.89	0.56	0.33	0.07	6.55	7.96	0.72	0.00
10 mg/kg																
Mean	5.94	8.02	15.45	43.88	54.75	19.29	35.23	988.69	17.69	17.58	7.60	0.75	23.45	66.48	2.11	0.00
SD	2.63	0.31	0.53	1.58	0.95	0.86	1.13	69.75	0.58	0.37	0.35	0.06	4.68	7.21	0.94	0.00
100 mg/kg																
Mean	4.94	8.06	15.46	43.75	54.34	19.20	35.33	983.17	18.13	17.54	7.62	0.75	21.41	71.81	1.84	0.00
SD	1.64	0.38	0.72	1.68	1.09	0.83	1.21	87.48	0.79	0.44	0.35	0.06	10.20	10.80	0.94	0.00
1,000 mg/kg																
Mean	6.57	8.15	15.56	43.64	53.55	19.08	35.65	984.64	17.71	17.74	7.73	0.76	18.59	73.57	1.89	0.00
SD	1.32	0.28	0.59	0.92	1.06	0.64	1.05	65.33	0.84	0.48	0.49	0.06	5.24	7.91	0.92	0.00

Female																
Control																
Mean	4.60	8.04	16.03	45.05	56.06	19.98	35.62	758.50	16.39	18.17	8.15	0.61	18.18	74.09	2.08	0.00
SD	1.63	0.32	0.55	1.57	0.74	0.61	0.99	167.94	0.70	1.18	1.13	0.15	11.32	14.09	1.26	0.00
10 mg/kg																
Mean	4.05	7.79	15.73	44.21	56.74	20.20	35.61	754.86	16.28	17.90	7.93	0.60	19.24	67.69	2.62	0.00
SD	1.70	0.39	0.75	1.80	0.68	0.82	1.26	65.17	0.69	0.44	0.47	0.06	7.72	7.75	1.52	0.00
100 mg/kg																
Mean	4.19	7.84	15.61	44.32	56.56	19.94	35.23	824.18	16.09	17.65	7.93	0.65	18.42	73.60	2.46	0.00
SD	2.11	0.26	0.67	1.30	0.79	0.53	0.85	59.44	0.96	0.48	0.28	0.05	6.92	10.55	1.91	0.00
1,000 mg/kg																
Mean	4.12	7.66	15.24	43.27	56.51	19.91	35.21	728.67	16.04	17.33	7.72	0.56	17.90	74.17	2.46	0.00
SD	0.95	0.35	0.72	1.74	1.23	0.84	0.87	72.59	0.76	0.60	0.35	0.05	9.72	11.57	1.96	0.00

WBC: white blood cell, RBC: red blood cell, HCT: hematocrit, MCV: mean corpuscular volume, MCH: mean corpuscular hemoglobin, MCHC: mean corpuscular hemoglobin concentration, PLT: platelet, RDW: red cell distribution width, PDW: platelet distribution width, MPV: mean platelet volume, PCT: plateletcrit, Neut: neutrophil, Lymph: lymphocyte, Eosi: eosinophil, Mono: monocyte.

**Table 2:** Sprague Dawley mean blood clinical chemistry value in chronic oral toxicity test.

Group	GLU (mg/dL)	BUN (mg/dL)	CREA (mg/dL)	CHOL (mg/dL)	TG (mg/dL)	URIC (mg/dL)	TP (g/dL)	ALB (g/dL)	GLOB (g/dL)	Bili-T (U/L)	AST (U/L)	ALT (U/L)	ALP (U/L)
Male													
Control													
Mean	137.56	23.21	0.66	230.29	192.29	1.64	9.43	4.80	4.63	0.19	99.29	68.76	80.29
SD	15.30	2.17	0.09	48.24	64.14	0.52	1.24	0.56	0.78	0.02	18.07	16.94	18.56
10 mg/kg													
Mean	155.22	23.73	0.66	256.08	210.08	1.82	9.26	4.62	4.64	0.17	95.73	68.29	77.38
SD	35.92	3.12	0.11	60.33	68.66	0.68	0.89	0.37	0.65	0.03	14.04	14.99	16.43
100 mg/kg													
Mean	162.46	24.98	0.70	264.50	194.92	2.18	9.89	5.01	4.88	2.17	100.20	69.98	81.50
SD	29.86	4.34	0.14	53.29	51.49	0.53	0.88	0.65	0.52	6.87	21.01	20.43	14.06
1,000 mg/kg													
Mean	182.29	24.81	0.60	279.82	211.91	2.73	9.23	4.61	4.62	0.15	80.11	58.26	80.18
SD	38.54	2.87	0.14	84.66	97.57	0.85	1.05	0.45	0.84	0.04	22.03	11.19	9.10

Female													
Control													
Mean	185.88	23.06	0.70	173.50	115.71	2.38	10.34	5.94	4.41	0.27	141.31	73.76	41.29
SD	41.89	2.89	0.08	31.71	20.01	0.81	0.83	0.44	0.48	0.06	19.97	14.02	7.28
10 mg/kg													
Mean	168.03	22.10	0.64	173.79	131.57	2.34	9.74	5.67	4.06	0.23	137.86	69.34	40.36
SD	35.19	2.41	0.10	35.43	26.26	0.79	1.11	0.55	0.66	0.06	29.51	18.77	11.04
100 mg/kg													
Mean	157.04	22.67	0.66	180.00	119.09	1.89	10.59	5.98	4.62	0.27	149.45	77.83	47.45
SD	23.06	3.57	0.09	48.49	30.03	0.46	1.09	0.60	0.59	0.09	28.34	15.41	13.38
1,000 mg/kg													
Mean	158.46	24.34	0.63	176.00	120.00	1.96	10.04	5.76	4.30	0.21	126.00	57.00	42.78
SD	27.49	4.15	0.13	36.24	36.48	0.68	1.13	0.49	0.77	0.04	39.37	21.07	23.87

GLU: glucose, BUN: blood urea nitrogen, CREA: creatinine, CHOL: cholesterol, TG: triglyceride, TP: total protein, ALB: albumin, GLOB: globulin, Bili-T: total bilirubin, AST: aspartate aminotransferase, ALT: alanine transaminase, and ALP: alkaline phosphatase.

**Table 3:** Prevalence of histopathological changes in the liver, heart, lung, spleen, and kidney of both sexes among 10, 100, and 1,000 mg/kg/day dosages and control group.

Histopathological changes/parameter	Prevalence (%) or mean score				*P* value
	Control	PN (mg/kg/day)			
		10	100	1,000	
Liver					
Focal lymphocyte aggregation (mild)	30 (6/20)	25 (5/20)	21 (4/19)	12 (2/17)	0.600
Bile duct dilatation (mild)	10 (2/20)	5 (1/20)	5 (1/19)	0 (0/17)	0.605
Microvesicular steatosis (mild)	10 (2/20)	15 (3/20)	0 (0/19)	6 (1/17)	0.357
Macrovesicular steatosis (mild)	15 (3/20)	20 (4/20)	11 (2/19)	6 (1/17)	0.619
Bile duct hyperplasia (mild)	0 (0/20)	5 (1/20)	16 (3/19)	18 (3/17)	0.181
Hepatic karyorrhexis (mild)	85 (17/20)	90 (18/20)	95 (18/19)	94 (16/17)	0.707
Heart					
Focal endocarditis (mild)	65 (13/20)	55 (11/20)	47 (9/19)	35 (6/17)	0.324
Focal coagulative necrosis (mild)	55 (11/20)	65 (13/20)	53 (10/19)	47 (8/17)	0.733
Lung					
Peribronchial cuffing (moderate)	100 (20/20)	95 (19/20)	100 (19/19)	94 (16/17)	0.533
Perivascular edema (moderate)	75 (15/20)	70 (14/20)	89 (17/19)	88 (15/17)	0.177
Perivascular cuffing (moderate)	100 (20/20)	100 (20/20)	89 (17/19)	100 (17/17)	0.104
Alveolar septal thickening (moderate)	100 (20/20)	100 (20/20)	100 (19/19)	100 (17/17)	—
Alveologenic edema (moderate)	50 (10/20)	65 (13/20)	74 (14/19)	76 (13/17)	0.307
Histiocytosis (mild)	40 (8/20)	35 (7/20)	26 (5/19)	24 (4/17)	0.678
Tunica medial hyperplasia (moderate)	80 (16/20)	90 (18/20)	89 (17/19)	88 (15/17)	0.768
Spleen	Normal finding				
Kidney					
Renal pelvis calcification (moderate)	**50 (10/20)**	0 (0/20)	0 (0/19)	0 (0/17)	**0.000**
Tubular/interstitial calcification (mild)	100 (20/20)	85 (17/20)	84 (16/19)	82 (14/17)	0.768
Transitional cell hyperplasia (moderate)	**35 (7/20)**	5 (1/20)	0 (0/19)	0 (0/17)	**0.002**
CPN (early stage)	50 (10/20)	45 (9/20)	42 (8/19)	41 (7/17)	0.947
Pigmented tubules (mild)	75 (15/20)	60 (12/20)	68 (13/19)	65 (11/17)	0.782
Interstitial nephritis (mild)	55 (11/20)	65 (13/20)	53 (10/19)	53 (9/17)	0.847
Mesangiolysis: Lt kidney	10.37 ± 2.49	10.68 ± 7.98	12.27 ± 4.26	**55.44 ± 22.2**	**0.000**
: mean score	1.51 ± 0.39	1.31 ± 0.25	1.37 ± 0.24	1.50 ± 0.08	0.388
Mesangiolysis: Rt kidney	10.63 ± 2.87	7.05 ± 4.32	17.05 ± 3.05	**44.58 ± 10.3**	**0.000**
: mean score	1.42 ± 0.31	1.27 ± 0.19	1.33 ± 0.23	1.53 ± 0.22	0.211

CPN: chronic progressive nephropathy, Lt: left, and Rt: right.
